# Oral Processing Studies: Why Multidisiciplinary?

**DOI:** 10.3390/foods9070875

**Published:** 2020-07-03

**Authors:** Susana Fiszman, Amparo Tarrega

**Affiliations:** Instituto de Agroquímica y Tecnología de Alimentos (IATA-CSIC), 46980 Valencia, Spain; atarrega@iata.csic.es

**Keywords:** oral processing, mastication, swallowing, temporal dynamic measurements, in-mouth sensory perception

## Abstract

When food is ingested, it remains in the mouth for a short period of time. Although this period is brief compared to the total food nutrient digestion and absorption time, it is crucially important as it is the first step in digestion. It is also very important that, while the food is in the mouth, it is perceived by the senses and then a decision is made on swallowing. Oral sensory perception is an integrative response, which is generated in very short time (normally a few seconds) from complex information gathered from multiple sources during mastication and swallowing. Consequently, food oral processing studies include many orientations. This Special Issue brings together a small range of studies with a diversity of approaches that provide good examples of the complexity and multidisciplinarity of the subject.

## 1. Introduction

From the moment a piece of food enters the mouth, it is continuously assessed to decide how to handle it in the next instant. This depends on the level of force at which it breaks, how it breaks, how the broken pieces behave, how many and how big they are, how much saliva they absorb, how easily they form a bolus, among other factors related to the series of actions which lead to the final swallow. Throughout this time span (only a few seconds), the senses of touch, smell, taste, and hearing collect data to form an integrative perception of that food.

Consequently, oral processing can be approached from several points of view, such as physiological, nutritional, psychological, metabolic, sensory, mechanical, and chemical, among others and, importantly, all of them can be studied with a sense of evolution and a sense of dynamics. 

One study in the present themed issue adopted an original sensory approach to wine [1]. It focused on “body”, a quality of wine that could be roughly defined as “how it feels inside the mouth”, which is constantly mentioned by experts, sommeliers, and consumers. A number of individual components of wine have previously been associated with “body” and their instrumental analysis has been correlated to sensory perception. In this study, several of these components and their combinations were tested [1], introducing saliva as a new variable, and instrumental and sensory analyses were performed and correlated to wine body perception.

An interesting mechanical approach was taken in an investigation of food for a population of advanced age [2]. Care food is normally designed to be broken easily in the mouth just by pressing it against the palate, without the participation of teeth. To follow the fracture patterns of the experimental food, the authors used an artificial tongue made of a soft material instead of the hard surfaces of a conventional texture analyzer. Video recordings of the food deformation and fracture indicated that the soft system mimics the natural oral processing of care food in a more reliable way.

Patterns of in vitro fragmentation were also studied in another paper [3] in the present themed issue, but through a different approach. In this case, the influence of the food matrix (rather than the composition) was investigated with oat biscuits. In vitro fragmentation of biscuits with the same composition differed depending on the morphology of the oat ingredient. In turn, these differences play a role in the level of starch hydrolysis in the oral phase.

Temporal aspects of oral perception dominate the three remaining papers. One studies the relative importance of several factors in the temporal aroma-compound release that takes place during eating [4]. These factors are the physicochemical properties of the food matrix, the characteristics of the aroma compounds, and oral physiological parameters, such as bite force, shearing angle, and salivary flow rate. All the oral parameters were measured using a chewing simulator and the aroma compound releases were followed in real time through atmospheric pressure chemical ionization mass spectrometry. Interesting differences in aroma release were found depending on the hydrophobicity of the volatile compound.

The second paper, dealing with the temporal aspects of food oral processing, studies the in-mouth perception of carrot purees designed for dysphagic patients, which were thickened with starch and xanthan gum, and their combinations [5]. Two temporal sensory techniques were used: Temporal Dominance of Sensations (TDS); Temporal Check-all-that-Apply questions (TCATA). “Grainy” was associated with the starch-thickened puree, while “smooth” and “slippery” were associated with xanthan gum. The oral perception of all the puree samples evolved from predominantly thick to adhesive, with a mouthcoating sensation towards the end of the evaluation. Both TDS and TCATA yielded similar results.

Finally, the third of these papers studies the temporal perception of the texture of complementary porridges for infants and young children used in low-income African communities [6]. This paper is very interesting not only technically but also because of its social dimension. The sensory quality of these porridges affects oral abilities, leading to malnutrition. The TCATA sensory method was used to assess indigenous and commercial porridges. The assessors used two different oral techniques: up and down movements to mimic babies’ mastication; normal movements, as in normal eating. The results reveal that the indigenous porridges were too thick and sticky, and not easy to swallow, even with a low solids content—especially using the Up-Down method. This highlights that texture improvement should be promoted for this complementary type of feeding.

The diversity of the approaches presented in “Contribution of Food Oral Processing” encourages an exploration of potential areas of collaboration which could give rise to a better understanding of the mechanisms involved in eating. The editors hope the readers will find this issue interesting and useful for inspiring future research.
